# DNA methylation footprints during soybean domestication and improvement

**DOI:** 10.1186/s13059-018-1516-z

**Published:** 2018-09-10

**Authors:** Yanting Shen, Jixiang Zhang, Yucheng Liu, Shulin Liu, Zhi Liu, Zongbiao Duan, Zheng Wang, Baoge Zhu, Ya-Long Guo, Zhixi Tian

**Affiliations:** 1 State Key Laboratory of Plant Cell and Chromosome Engineering, Institute of Genetics and Developmental Biology, Chinese Academy of Sciences, Beijing, 100101 China; 2 State Key Laboratory of Systematic and Evolutionary Botany, Institute of Botany, Chinese Academy of Sciences, Beijing, 100093 China; 3 University of Chinese Academy of Sciences, Beijing, 100039 China

**Keywords:** DNA methylation, Domestication, Soybean

## Abstract

**Background:**

In addition to genetic variation, epigenetic variation plays an important role in determining various biological processes. The importance of natural genetic variation to crop domestication and improvement has been widely investigated. However, the contribution of epigenetic variation in crop domestication at population level has rarely been explored.

**Results:**

To understand the impact of epigenetics on crop domestication, we investigate the variation of DNA methylation during soybean domestication and improvement by whole-genome bisulfite sequencing of 45 soybean accessions, including wild soybeans, landraces, and cultivars. Through methylomic analysis, we identify 5412 differentially methylated regions (DMRs). These DMRs exhibit characters distinct from those of genetically selected regions. In particular, they have significantly higher genetic diversity. Association analyses suggest only 22.54% of DMRs can be explained by local genetic variations. Intriguingly, genes in the DMRs that are not associated with any genetic variation are enriched in carbohydrate metabolism pathways.

**Conclusions:**

This study provides a valuable map of DNA methylation across diverse accessions and dissects the relationship between DNA methylation variation and genetic variation during soybean domestication, thus expanding our understanding of soybean domestication and improvement.

**Electronic supplementary material:**

The online version of this article (10.1186/s13059-018-1516-z) contains supplementary material, which is available to authorized users.

## Background

Agriculture feeds more than seven billion people on this planet [1]. In the development of agriculture, domestication is regarded as one of the most important events [2]. To improve the growth and performance of cultivated species in agricultural environments, humans carried out artificial selection on wild species during the process of domestication. The selection changed various traits to optimize cultivated species, such as higher yield, larger seeds, reduced seed dispersal, and reduced seed dormancy [3]. Genetically, domestication is a process of modification of genomic diversity in the cultivated populations [4]. Identification of the corresponding loci or genes relevant to domestication will accelerate future crop improvement [3, 5].

Benefiting from the rapid development of next-generation sequencing technology, various investigations of artificial selection at the genome level during plant domestication have been performed in different species, which identified a number of domestication sweeps and provided valuable resources for genomics-enabled improvements in crop breeding [6–17]. However, most of these investigations focused on genetic variation. Besides genetic variation, epigenetic variation also plays essential roles in diverse biological processes [18–20]. Epigenetic modifications can create epialleles that can be inherited independently [21]. Furthermore, compared with genetic changes, epigenetic variation evolves more quickly [22, 23]. The inheritance of epigenetic variation may partially explain the missed heredity in genome-wide association studies (GWAS) of genetic variation [24]. Therefore, epigenetic variation represents an important source of natural variation that could be used in plant-breeding programs [20, 22, 25–27].

DNA methylation is one of the most extensively studied epigenetic modifications in plants [28, 29]. DNA methylation can influence transcriptional activity [29–33], morphological development [34–38], and agronomic trait formation [37, 39, 40]. In addition, it also plays an important role in evolution [41, 42]. Population analyses have demonstrated that DNA methylation varies among the individuals within a species [43, 44] and that these variations could lead to extensive phenotypic variations [31, 45, 46], such as biomass [47], energy use efficiency [25], disease resistance [48], and environmental adaptation [44, 49, 50]. It has been demonstrated that domestication may alter DNA methylation profiles [43, 51]. In a recent study, DNA methylation variation in *CONSTANS*-*LIKE* (*COL*) genes was found to be responsible for the loss of photoperiod sensitivity during cotton domestication [52]. These studies suggest that DNA methylation variation is an important component of artificial selection in crop domestication beyond genetic variation, and thus, is crucial in plant breeding and agriculture [27].

Soybean (*Glycine max* [L.] Merr.) is one of the most important crops and accounts for more than half of global oilseed production [53]. Cultivated soybean was domesticated from wild soybean (*G. soja* Sieb. & Zucc.) in China 5000 years ago [53–55]. Compared to wild soybean, cultivated soybean exhibits significant changes in diverse morphological characteristics [16, 53]. Comprehensive resequencing analyses of wild soybeans, landraces, and cultivars have clarified the demographic history and identified the genetic regions that experienced selective sweeps during soybean domestication and improvement [7, 16, 56]. Furthermore, the integration of a GWAS of domestication traits with previous quantitative trait loci (QTL) analyses revealed that some of these selective sweeps may be associated with the increase of oil content in cultivated soybeans [16]. Whole-genome bisulfite sequencing (WGBS) of 83 soybean recombinant inbred lines (RILs) revealed that the observed DNA methylation variation was heritable [57]. Thus far, the importance of epialleles in soybean domestication and improvement and its relationship with genetic selection was largely unknown. Genome-wide study of epigenetic variants, together with the previous genetic analyses, will enhance our understanding of soybean domestication and improvement.

Here, we generated single-base-resolution methylomes of 45 soybean accessions, including wild soybeans, landraces, and cultivars. Through a comprehensive investigation of methylation variation, we identified 5412 differentially methylated regions (DMRs) during soybean domestication and improvement. The genetic diversity between DMRs and selective genetic regions are significantly different. Moreover, we discovered that genes related to carbohydrate metabolism were significantly enriched in the DMRs that were not associated with any genetic variation, indicating that the methylation variation may play an important role independently from that of genetic selection in soybean domestication.

## Results

### Differentially methylated regions (DMRs) during soybean domestication and improvement

**Fig. 1:**
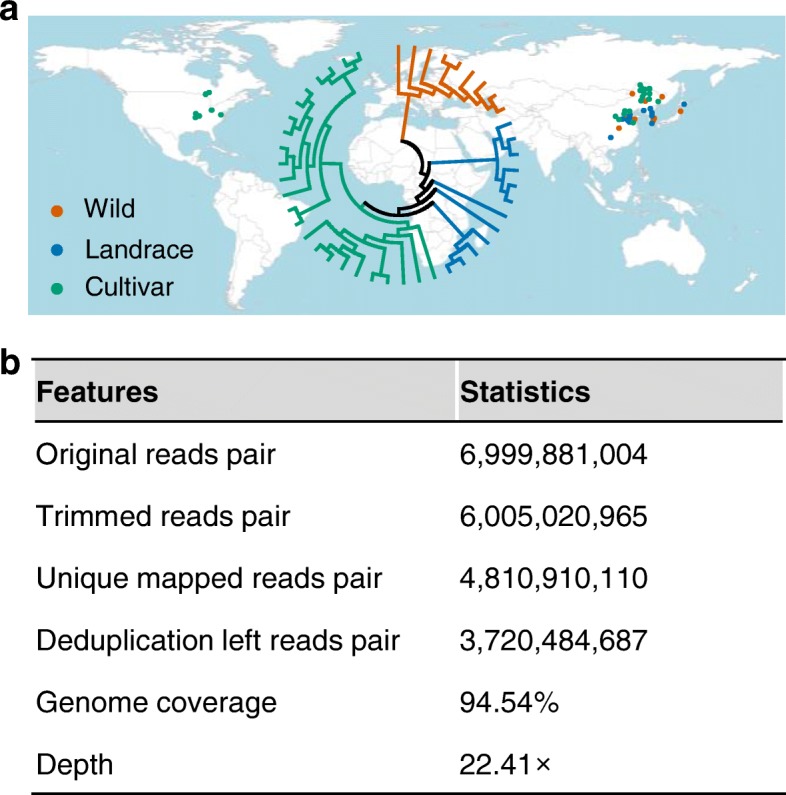
Accession information and methylation sequencing. **a** Geographical distribution and phylogenetic tree of the 45 sequenced accessions. **b** Summary of whole-genome bisulfite sequencing. Statistics for reads pairs were the sum of all sequenced accessions, statistics for genome coverage and depth were the average of all sequenced accessions

To uncover DNA methylation changes during soybean domestication and improvement, we performed WGBS on 45 representative accessions from our previous studies [16, 58], including nine wild soybeans, 12 landraces, and 24 cultivars (Fig. 1a; Additional file 1: Table S1). In total, > 1919 Tb sequences were generated. To exclude the effects of nucleotide variation across these accessions in DNA methylation analysis, we performed resequencing for these accessions on an Illumina HiSeq sequencer with an average sequencing depth of > 20× (Additional file 2: Table S2). The resequencing reads were mapped to the cultivated soybean Williams 82 reference genome. Single-nucleotide polymorphisms (SNPs) from individual accession (Additional file 2: Table S2) were used to replace the corresponding nucleotides in the reference genome to generate a pseudo-reference genome for each accession, following the previous method [50] (see “Methods”).

For methylation analysis, after trimming the adapters and low-quality bases, the remaining WGBS reads were mapped to the soybean pseudo-reference genome of each accession (Additional file 3: Figure S1). After removing the duplicated reads, a total of 3720 million uniquely mapped read pairs, which covered 94.54% of the cultivated soybean Williams 82 reference genome with an average depth of 22.41×, were retained (Fig. 1b; Additional file 4: Table S3). In plants, DNA methylation occurs in three contexts: CG; CHG; and CHH (H = C, A, or T) [59]. After removing cytosine sites with sequencing depths < 4 and performing binomial tests using the unmethylated chloroplast genome as control (Additional file 3: Figure S1), a total of 16,836,566 methylated CGs (mCG) (52.7% of all CGs), 16,333,099 mCHGs (41.3% of all CHGs), and 11,678,796 mCHHs (4.4% of all CHHs) were identified (Additional file 5: Table S4). These results represented a similar proportion of methylated cytosines to that was found in a previous report from soybean RILs [57].

**Fig. 2:**
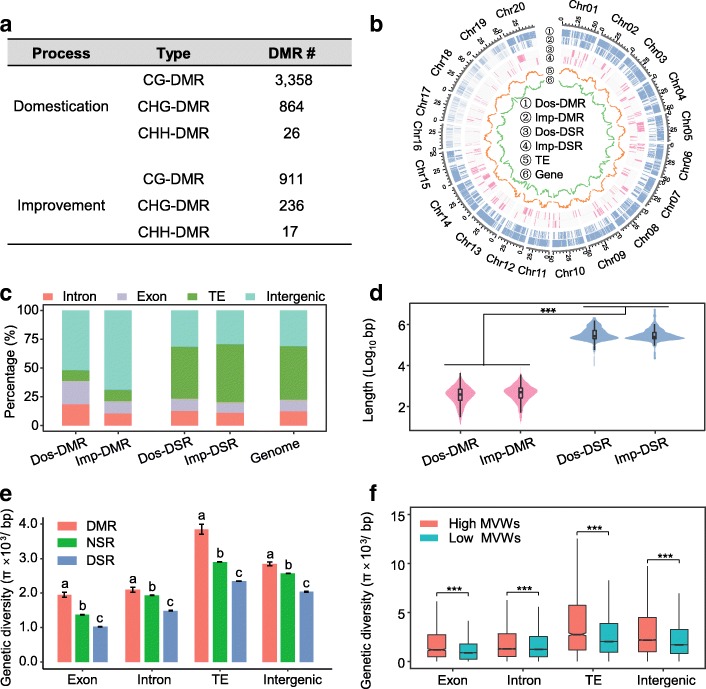
Differentially methylated region (DMR) detection and comparison to DNA sequence regions under selection (DSRs). **a** DMRs detected in soybean domestication and improvement. **b** Genome-wide distributions of DMRs and DSRs. **c** Genomic compositions of DMRs and DSRs. TE regions were defined as regions masked by RepeatMasker using soybean annotated TEs as the library. **d** Length comparison between DMRs and DSRs. ***p < 0.001 by ANOVA. **e** Genetic diversity comparisons between DMRs, DSRs, and non-selected regions (NSRs) from different genomic regions. ANOVA were performed for each genomic region. Different letters at the top of each column indicate significant differences by ANOVA (*p* < 0.001). **f** Genetic diversity comparisons between high methylation variation windows (MVWs) and low methylation variation windows for different genomic regions. ***p < 0.001 by t-test

To examine the DNA methylation variation during soybean domestication (wild soybeans versus landraces) and improvement (landraces versus cultivars), we identified DMRs between the different populations according to a previous method [50, 60, 61]. In total, 4248 DMRs were identified in the process of soybean domestication (termed Dos-DMR in this study), including 3358 CG-DMRs, 864 CHG-DMRs, and 26 CHH-DMRs. Compared with domestication, fewer DMRs were identified in the improvement process (termed Imp-DMR in this study), which amounted to 1164 DMRs, including 911 CG-DMRs, 236 CHG-DMRs, and 17 CHH-DMRs (Fig. 2a; Additional file 6: Table S5).

Compared to the DNA sequence regions under selection (termed DSRs; Dos-DSR for domestication and Imp-DSR for improvement in this study) that have previously been identified [16], DMRs exhibited different characters. The DMRs were distributed more evenly across the genome (Fig. 2b) and fewer DMRs than DSRs were located in transposable element (TE) regions (Fig. 2c). In addition, the average length of DMRs was significantly shorter than that of DSRs (ANOVA, *p* < 2.2e-16) (Fig. 2d). However, no significant differences in length were found between Dos-DMRs and Imp-DMRs (ANOVA, *p* = 1.000) or between Dos-DSRs and Imp-DSRs (ANOVA, *p* = 0.058) (Fig. 2d).

### DMRs exhibited higher genetic diversity

Generally, genetic diversity is reduced in domesticated lines because of genetic bottleneck effect during domestication [62]. To investigate the effects of DNA methylation variation on the genetic diversity of DMRs, we compared the genetic diversity among DMRs, DSRs, and non-selected regions (termed NSRs in this study, which are the genomic regions outside the DMRs and DSRs). Previous studies have demonstrated that TEs usually exhibit high genetic variations in a population [63, 64]. In the soybean genome, the average genetic diversity (represented by π) in TE regions is higher than that in intergenic and genic regions (Additional file 3: Figure S2). Because the genomic compositions of DMRs and DSRs are significantly different (Fig. 2c), to eliminate their effects, we investigated the genetic diversities of DMRs, DSRs, and NSRs separately in different genetic regions. The results showed that DSRs exhibited lower genetic diversity than NSRs in diverse contexts (Fig. 2e). In contrast, the genetic diversity in DMRs was higher than that of NSRs (Fig. 2e). When the genetic diversity was investigated in individual populations, similar patterns were observed, particularly for the exon and TE regions (Additional file 3: Figure S3).

One possible reason for the higher genetic diversity in DMRs may be directly resulted from the variation of methylation level, which means that, for a specific region, the increase/decrease of its methylation level in a particular population could affect its mutation rate. To examine this possibility, we divided the DMRs into two groups: decreased-DMRs (the methylation level in the selected population is decreased, i.e. landraces compared to wild soybeans and cultivars compared to landraces) and increased-DMRs (the methylation level in the selected population is increased). If the mutation rate could be affected by methylation level, we expected to see a consistent pattern of genetic diversity changes in individual groups and to see a correlation between genetic diversity change and DMR level. However, a mixture pattern of increasing/decreasing/unchanging genetic diversity was observed in both of the decreased-DMRs and the increased-DMRs, either from domestication or from improvement processes (Additional file 3: Figure S4). Pearson correlation analysis between genetic diversity variation and DMR level in each category also suggested that they were inconspicuously correlated (Dos-increase: *r* = 0.089, *p* = 0.003; Dos-decrease: *r* = 0.062, *p* = 0.001; Imp-increase: *r* = 0.112, *p* = 0.011; Imp-decrease: *r* = − 0.015, *p* = 0.697). Another possible reason for the higher genetic diversity in DMRs could be associated with the process of domestication and improvement directly, which increased the mutation rate of DMR in the selected population along with its methylation level variation. In this case, we would expect to see increased genetic diversity in the selected populations (i.e. landraces compared to wild soybeans and cultivars compared to landraces). However, the landraces exhibited significantly lower genetic diversity than the wild soybeans and the cultivars exhibited significantly lower genetic diversity than the landraces (t-test, *p* < 0.001; Additional file 3: Figure S5), suggesting that the higher genetic diversity in DMRs did not come directly from the process of domestication and improvement.

To further determine whether methylation changes were indeed associated with mutation rate, we calculated the differences of methylation levels between wild soybeans and landraces and between landraces and cultivars in contiguous 500-bp (approximately the average DMR length) windows across the soybean genome. Meanwhile, genetic diversity was also investigated in these windows. The windows were divided into two groups: windows with relatively high methylation variation (> 0.4, same criterion of DMR) and windows with relatively low methylation variation (< 0.4, same criterion of DMR). Then, the average genetic diversity levels of the windows in each group were compared for the different genomic regions. We observed that the windows with high methylation variation exhibited higher genetic diversity than the windows with low methylation variation (Fig. 2f). Taken together, these results indicated that higher genetic diversity might be an inherent character of regions with higher methylation variation.

### Characterization of different DMR contexts

**Fig. 3:**
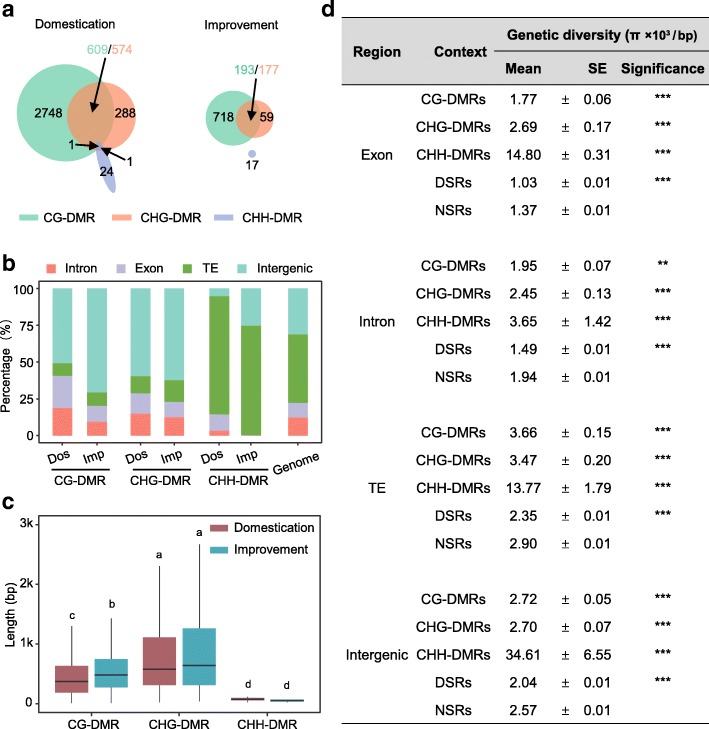
Characterization of DMRs in different cytosine contexts. **a** Overlapping DMRs from different methylation contexts in the domestication and improvement processes. **b** Genomic compositions of different DMRs types. **c** Length comparisons between different DMR types. Different letters at the top of each column indicate significant differences by ANOVA (*p* < 0.001). **d** Genetic diversity comparisons among different DMR types from different genomic regions. Mann–Whitney U-test was performed between NSR and other DMR types for different genomic regions. ***p < 0.001, **p < 0.01

In our analyses, although the number of methylated CG cytosine sites was equal to that of CHG and approximately 1.5 times higher than that of CHH in the populations (Additional file 5: Table S4), many more CG-DMRs were identified than CHG-DMRs, and only a few CHH-DMRs were detected in soybean domestication and improvement (Fig. 2a). Further investigation revealed that more than half of the CHG-DMRs (574 of the 864 Dos_CHG-DMRs and 177 of the 236 Imp_CHG-DMRs) overlapped with CG-DMRs. However, few CHH-DMRs were found to overlap with regions of the other two contexts (Fig. 3a). Only a small number of DMRs for individual methylation contexts were shared by the two selection processes, domestication and improvement (Additional file 3: Figure S6), which is consistent with the patterns found in genetic selection sweep analyses [16]. The CG-DMRs and CHG-DMRs were further classified into three groups based on their positional relationships: unique CG-DMRs (termed u-CG-DMRs in this study), unique CHG-DMRs (termed u-CHG-DMRs in this study), and overlapping CG-DMRs and CHG-DMRs (termed o-CG/CHG-DMRs in this study). Interestingly, we found that the variations in CG and CHG methylation exhibited the same trends in the o-CG/CHG-DMRs for both the domestication (Additional file 3: Figure S7a) and improvement processes (Additional file 3: Figure S7b). Furthermore, the correlation between CG and CHG methylation in o-CG/CHG-DMRs was much higher than that in u-CG-DMRs and u-CHG-DMRs (Additional file 3: Figure S7c and d), suggesting that the CG and CHG methylation in o-CG/CHG-DMRs may evolve together somehow.

Subsequently, we compared the characters among DMRs of different contexts and found that CHH-DMRs were significantly different from CG-DMRs and CHG-DMRs. A higher proportion of CHH-DMRs was found in TE regions, while more CG-DMRs occurred in genic regions (Fig. 3b), consistent with previous observations in *Arabidopsis* [44, 50, 65]. In addition, we found that the average length of the CHH-DMRs was significantly shorter than that of DMRs of the other two contexts (Fig. 3c). However, the average length of o-CG/CHG-DMRs was longer than that of u-CG-DMRs and u-CHG-DMRs (Additional file 3: Figure S8).

DNA methylation in each context (CG, CHG, and CHH) is linked to specific biological functions and is primarily established and maintained by distinct DNA methyltransferase pathways [59, 66, 67]. Generally, CG-DMRs reflect variable CG gene body methylation [50]. Given the above analysis suggested that an association might exist between genetic diversity variations and DMRs (Fig. 2e and f), to determine whether this association consistently existed in different cytosine contexts or was only present in a specific cytosine context, we investigated the genetic diversity of CG-DMRs, CHG-DMRs, and CHH-DMRs. Our results demonstrated that the π value of each cytosine context of DMRs was significantly higher than those of NSRs and DSRs, with the highest in CHH-DMRs (Fig. 3d), confirming that higher genetic diversity was a common character for all types of DMRs.

### Genetic variations contributing to DMRs

Local genetic variations, including TE insertion/deletion [59, 68, 69], SNPs [43, 50], and 24 nt small interfering RNA (siRNA) expression variation [70, 71], could influence methylation. To identify the local genetic variations that might be associated with our detected DMRs, we performed association analyses between methylation variation and three forms of genetic variation: siRNA expression variation; TE presence/absence; and local SNPs.

**Fig. 4:**
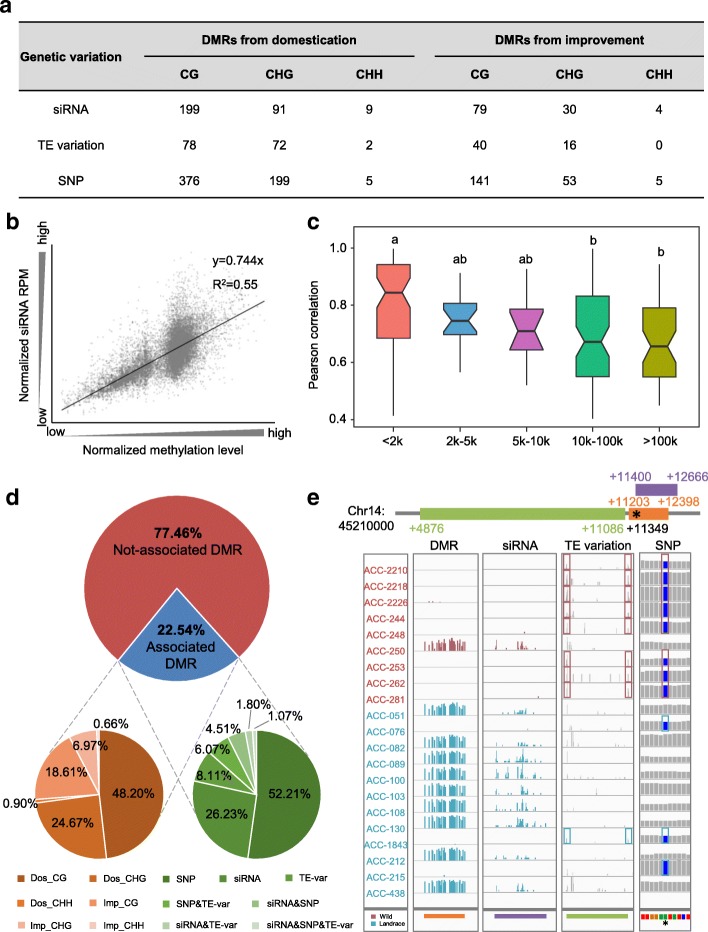
Local association study between DMRs and genetic variations. **a** Summary of the associations between DMRs and local siRNA expression variation, TE variation and SNPs. **b** Plot of methylation levels (*x-axis*) and siRNA expression values (*y-axis*). Methylation level and siRNA RPM were mean-centered and normalized. **c** Correlation between DMR methylation and TE variant state at different distances. The DMR/TE variation pairs were divided into five groups according to the distance between DMR and TE variant. Different letters at the top of each column indicate significant differences by ANOVA (*p* < 0.001). **d** The proportion of DMRs associated and not associated with local genetic variations (*top*) and the proportion of different DMR types (*bottom left*) and different genetic variation combinations (*bottom right*) for locally associated DMRs. **e** An example (Dos_CHG-DMR, Chr14:45,221,203–45,222,398) DMR that was simultaneously associated with local siRNA expression, TE variant, and SNP sites. Rectangles in the TE variant panel indicate reads supporting the TE variant and rectangles in the SNP panel indicate SNP sites

For the siRNA analysis, we performed small RNA sequencing (smRNA-seq) using the same samples for WGBS. A total of 40,575 24 nt siRNAs were identified, among which 1401 siRNAs were physically overlap with the DMRs we identified. Then, we performed a correlation analysis between the methylation variations and siRNA expression variations for each overlapping siRNA and DMR pair (see “Methods”). We found that the methylation changes in 412 DMRs (Fig. 4a) were significantly correlated with expression variations in their overlapping siRNAs (Fig. 4b; Additional file 3: Figure S9; Additional file 7: Table S6).

For the TE analysis, we investigated TE variants at a genome-wide level, referring to a previous methodology [72] and using the resequencing data from the 45 accessions. A total of 5663 TE variants were identified in the population. Then, the association between the methylation changes of each accession in each individual DMRs and the presence/absence of its closest TE were analyzed. The results indicated that the methylation changes in 208 DMRs were associated with TE variants (Fig. 4a; Additional file 8: Table S7). Moreover, TE variants at shorter distances exhibited higher association values than those at longer distances (Fig. 4c), suggesting that distance from a TE insertion or deletion influenced the methylation divergence level. Previous studies have suggested that indels can generate higher mutation rates [73–75]. Our analyses showed that the genetic diversity of DMRs associated with TE variants was higher than that of DMRs without TE variants (Additional file 3: Figure S10), suggesting that TE variation was one reason for the higher genetic diversity in DMRs (Fig. 2e). To identify local SNPs that might contribute to the DMRs, we performed a local association study based on a previously reported method [43]. We determined that the methylation changes of 779 DMRs might be associated with local SNPs (Fig. 4a; Additional file 9: Table S8).

Taken together, the association analyses of siRNA expression, TE variants, and local SNPs could explain the methylation variations of 1370 DMRs (22.54% of the total DMRs). The majority of these DMRs that were associated with genetic variations were CG-DMRs and CHG-DMRs (Fig. 4d). Consistent with the similar variation pattern between CG and CHG methylation in o-CG/CHG-DMRs (Additional file 3: Figure S7), we found that for those CG-DMRs and CHG-DMRs pairs in o-CG/CHG-DMRs that could detect association factors, a large proportion of them (189 of 263 pairs in domestication and 60 of 79 pairs in improvement) shared the same association genetic factors (Additional file 3: Figure S11). Beside these o-CG/CHG-DMRs, most of other DMRs were associated with distinct and independent genetic variations. Approximately 13.45% of DMRs were found to be affected by multiple factors and 1.07% were even simultaneously associated with siRNAs, TEs, and SNPs (Fig. 4d). For instance, the methylation levels of different accessions in the DMR located on chromosome 14 were significantly associated with siRNA expression, TE, and SNP variations (Fig. 4e).

### Genes from “pure DMRs” enriched in carbohydrate metabolism pathways

A primary goal of DMR analysis is to identify “pure epialleles” that are independent of genetic variation [29]. Such “pure epialleles” are an important source of phenotypic variation [21, 42]. In our analysis, 22.54% of DMRs were found to associate with local genetic variations; however, 77.46% of DMRs remained unexplained by these genetic variations (Fig. 4d). The DMRs that did not associate with any genetic variation were considered as “pure DMRs.”

Subsequently, we turned to investigate the genes that were located at these “pure DMRs.” Following the above classification, we also divided these “pure CG-DMRs” and “pure CHG-DMRs” into “pure u-CG-DMRs,” “pure o-CG/CHG-DMRs,” and “pure u-CHG-DMRs.” The genes overlapping with each of these “pure DMR” contexts were subjected to Gene Ontology (GO) and Kyoto Encyclopedia of Genes and Genomes (KEGG) enrichment analyses (Additional file 10: Table S9). We found that enriched categories were identified only in the genes from pure Dos-DMRs, not from the pure Imp-DMRs. In addition, for the pure Dos-DMRs, enrichments were only identified in the genes located in “pure u-CG-DMRs” and “pure o-CG/CHG-DMRs.” The biological process categories for these enriched genes were mainly macromolecule modification, protein modification process, and cellular process and the molecular function categories mainly include nucleotide binding, kinase activity, catalytic activity, transferase activity, and hydrolase activity (Additional file 3: Figure S12).

**Fig. 5:**
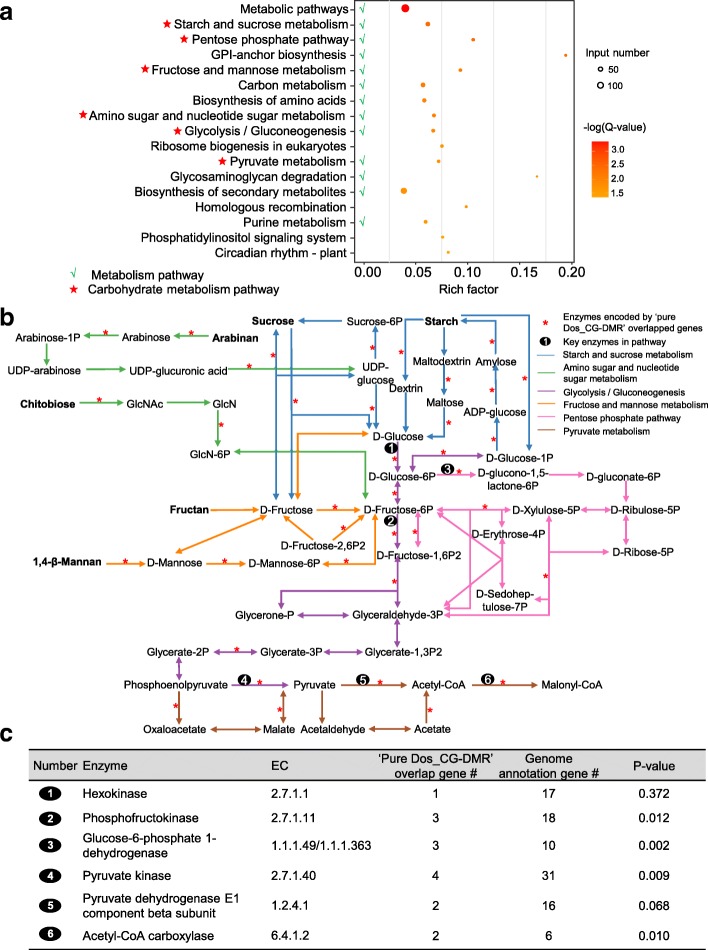
KEGG enrichment analysis of “pure Dos_CG-DMR” overlapping genes. **a** The pathways significantly enriched for “pure Dos_CG-DMR” overlapping genes. Pathways that contained > 5 overlapping genes with enrichment q-values < 0.05 were considered significantly enriched. **b** An integrated carbohydrate metabolism pathway composed of pathways enriched in “pure Dos_CG-DMR” overlapping genes. **c** Genome enrichment of six key enzymes in carbohydrate metabolism pathways. The background for “pure Dos_CG-DMR” overlapping genes was 1503 and that for genome annotation genes was 55,583; enrichment was analyzed by Fisher’s exact test

The KEGG analysis indicated that the genes overlapped with “pure CG-DMRs” in domestication were enriched in 17 pathways (Fig. 5a). Interestingly, 13 of these 17 pathways were related to metabolism. Moreover, six pathways belonged to carbohydrate metabolism, including starch and sucrose metabolism, pentose phosphate pathway, fructose and mannose metabolism, amino sugar and nucleotide sugar metabolism, glycolysis/gluconeogenesis, and pyruvate metabolism (Fig. 5a). Further investigation demonstrated that 62 “pure Dos_CG-DMR” overlapping genes were distributed throughout these carbohydrate metabolism processes (Fig. 5b; Additional file 11: Table S10). Six enzymes, including hexokinase, phosphofructokinase, glucose-6-phosphate 1-dehydrogenase, pyruvate kinase, pyruvate dehydrogenase E1 component beta subunit, and acetyl-CoA carboxylase, have been reported to play key roles in the glycolysis/gluconeogenesis, pentose phosphate pathway, and pyruvate metabolism, which are central pathways of carbohydrate metabolism [76–79]. The genes encoding these six enzymes were all found in “pure Dos_CG-DMRs” and four of them (phosphofructokinase, pyruvate kinase, glucose-6-phosphate 1-dehydrogenase, and acetyl-CoA carboxylase) were enriched (Fig. 5c).

## Discussion

DNA methylation is universally distributed across the genomes of most species [80]. Previous studies have indicated that DNA methylation can be responsive to climate change [22] and plays an important role in certain developmental processes [18]. Epigenetic diversity represents an essential source of natural variation that should be considered in plant-breeding programs [20, 22, 27]. However, the contribution of natural epigenetic variation to phenotypic variation remains enigmatic due to the relative lack of characterized natural epialleles [81–83].

Studies have demonstrated that, as genetic variation, epigenetic variation is heritable [57]. Mounting evidence indicates that a significant degree of variation in DNA methylation is genetically controlled [29]. However, the association degree between DNA methylation and genetic variation may vary in different species or in analyses of different populations [84]. For instance, an analysis of a large collection of Swedish *Arabidopsis* revealed that approximately 18% of DMRs were associated with genetic variants [61], whereas an early study of 152 methylomes in *Arabidopsis* from throughout the Northern Hemisphere suggested that the variation in 35% of DMRs could be explained by genetic variation [44]. In our analysis, we determined that approximately 22% of DMRs were associated with genetic variation (Fig. 4d). This number is much lower than that of a previous study using soybean RILs [57]. Most probably, the low association proportion in our study than that from RILs might be resulted from the more divergent natural population we used, including wild soybeans, landraces, and cultivars. A population with closer genetic relationships might show a higher correlation between genetic and epigenetic variations. This was in agreement with the study of North American *Arabidopsis* accessions that with close genetic relationships (more like RILs) revealed approximately 90% genotype–epigenotype associations [85], which is much higher than that in the natural population [44, 61]. Similarly, an analysis of maize RILs revealed that more than half of DMRs were associated with local genetic variants [43]. The variation in association degree between genetic variation and epigenetic variation from natural and closely genetically related populations of the same species may provide a clue that epigenetic variation is heritable independent from genetic variation.

Previous studies have suggested that epigenetic polymorphisms evolve faster than that of DNA sequences in the genome [23, 74, 86, 87]. Interestingly, our results demonstrated that DNA sequence diversity in DMRs was higher than that in other regions (Fig. 3d). Moreover, the regions with higher methylation variation among the population had higher genetic diversity than those with lower methylation variation (Fig. 2f). Although we could not fully explain how a high mutation rate was associated with high methylation variation, our analysis revealed that one reason might come from TE polymorphisms (Additional file 3: Figure S10), indicating that structural variations or indels may play important roles not only in the genome sequence mutation rate [73–75] but also in that of DNA methylation.

Plant domestication has been performed for thousands of years; this process has shaped plants for better growth and performance [3]. A comprehensive understanding of the mechanisms underlying agronomic traits is essential for generating better crop and breeding methodologies [19]. As a heritable genomic resource [80] that plays important roles in diverse developmental processes [18, 22], DNA methylation should also have undergone artificial selection during crop breeding. An interesting experiment by Haubena et al. [25] indicated the important role of epigenetic selection in the improvement of canola (*Brassica napus*). They performed recursive selection for respiration intensity and energy use efficiency (factors directly related to yield) on an isogenic doubled haploid line and found that three to five rounds of selection were sufficient to generate lines with distinct yield. However, these lines were found to be genetically identical but carried global epigenetic differences. Furthermore, both the agronomic traits and the DNA methylation patterns of the selected lines were heritable. A recent study in cotton also suggested that DNA methylation variations in several key genes were responsible for the loss of photoperiod sensitivity during cotton domestication [52].

Interestingly, our analysis demonstrated that genes related to metabolism exhibited significant DNA methylation level variation during soybean domestication, particularly genes related to carbohydrate metabolism (Fig. 5). Compared with their wild forms, cultivated soybeans exhibit significantly higher biomass, yield [3], and oil content [16]. Carbohydrate metabolism is an indispensable basis of yield and is also known to be related to fatty acid biosynthesis. Therefore, the significant DNA methylation level variation of metabolism-related genes during soybean domestication may be related to biomass and yield improvement or to high oil content. For instance, acetyl-CoA carboxylase catalyzes acetyl-CoA to form malonyl-CoA and malonyl-CoA is the basis for fatty acid biosynthesis [79]. In addition, the genes encoding three enzymes in fatty acid biosynthesis (malonyl-CoA-acyl carrier protein transacylase-like, long chain acyl-CoA synthetase, and 3-ketoacyl-CoA synthase) were all located in the DMRs, indicating that DNA methylation variation during domestication may be related to oil content.

The relationship between gene expression level and DNA methylation is complex. Previous studies have suggested that DNA methylation can influence transcriptional activity [29–33]. However, analyses at the genome-wide level in maize revealed that only approximately 20% of genes with qualitative (on-off) transcriptional differences were associated with DMRs; little association was identified between the expression of genes with quantitative transcriptional differences and DMRs [88]. Similarly, a recent study of > 1000 *Arabidopsis* accessions also suggested that gene body methylation does not have a major role in shaping transcriptional variation [50]. To determine whether methylation variation affected gene expression in these “pure DMRs,” we performed RNA-seq using the same samples used for WGBS. The transcriptional profiling analysis indicated no clear correlation between methylation changes and the transcriptional variation of the genes in these “pure DMRs” (Additional file 3: Figure S13). Therefore, the variation of DNA methylation at these enriched genes may not relate to changes in their expression.

The GO and KEGG enriched genes in our study all came from CG-DMRs involved in the domestication process. No significant enrichment was identified in the improvement process or in other methylation contexts. This result may have arisen because GO and KEGG annotations are confined to genes and more Dos_CG-DMRs were found in the genic regions than in other DMR contexts. Interestingly, in addition to the genic regions, a large proportion of DMRs were located in the intergenic regions (Fig. 2b). Long intergenic non-coding RNAs (lincRNAs) are found to play important roles in essential biological processes and a large number of lincRNAs exist in the intergenic regions of plant genomes [89]. The higher ratio of DMRs in the intergenic regions provides a clue that DNA methylation variation of the lincRNAs in these regions may be important, a hypothesis that should be further dissected. However, due to the limited characterization of lincRNAs in soybean, we could not perform further functional prediction of these elements. With the progress in the plant ENCODE (Encyclopedia of DNA Elements) project [90], we may be able to examine more clearly the role of epigenetic variation in crop domestication and improvement.

## Conclusions

Epigenetic variations play important roles in certain biological processes. Investigation of the contribution of epigenetic variation to plant domestication clarifies our understanding of domestication and will facilitate future crop breeding. Through a methylomic analysis of 45 soybean accessions, we found that DMRs exhibited characters distinct from those of genetic selection and that CG-DMRs that did not associate with genetic variations during soybean domestication could be correlated with carbohydrate metabolism. This study provides a valuable map of DNA methylation variation during soybean domestication and improvement.

## Methods

### Plant materials

All 45 soybean accessions were grown during the growing season of 2015 at the Beijing experimental station of the Institute of Genetics and Developmental Biology, Chinese Academy of Sciences. For each accession, the apical buds from 30 independent lines were collected at the stage of full true leaf expansion. The samples from each accession were mixed together for DNA and RNA extraction. DNA and RNA were isolated using a DNA/RNA isolation kit (Tiangen, Beijing, China) according to the manufacturer’s protocol.

### Library construction and sequencing

WGBS libraries were prepared according to the protocol described in a previous report [91]. The libraries for DNA-seq, RNA-seq, and small RNA-seq were prepared following the manufacturer’s instructions (Illumina Inc., San Diego, CA, USA). WGBS and DNA-seq libraries were sequenced on the Illumina HiSeq 2500 (125 bp paired-end reads) and Illumina HiSeq X10 (150 bp paired-end reads) platforms. The RNA-seq and small RNA-seq libraries were sequenced on the Illumina HiSeq 2500 platform. For the WGBS libraries, the sequence reads were mapped to the naturally unmethylated chloroplast genome of soybean using Bismark (ver. 0.14.5) [92] to evaluate the bisulfite non-conversion rate. The libraries with non-conversion rates < 1% were retained for further analysis.

### Resequencing analysis

Resequencing read mapping and SNP calling were performed as described previously [16] with the soybean reference genome v275 [93]. In brief, the SNPs were first called with GATK (ver. 3.1.1) [94] and SAMtools (ver. 0.1.19) [95] independently; then, the common sites identified by both methods were retained for pseudo-reference genome production. The phylogenetic tree was constructed by SNPhylo (ver. 20,160,204) [96]. The SNPs were filtered with linkage disequilibrium (LD) setting to 0.25 and the remaining SNPs were used to produce the maximum likelihood tree. The genetic diversity (π value) for each SNP was calculated using the formula introduced by Nei and Li [97].

### WGBS analysis

Adapters and low-quality bases in the WGBS reads were first trimmed by Trimmomatic (ver. 0.36) [98] using the following parameters: adapter.fa:2:40:15; LEADING:30; HEADCROP:6; TRAILING:30; SLIDINGWINDOW:4:15; AVGQUAL:30; and MINLEN:100. Subsequently, the trimmed reads were unique mapped to each corrected pseudo-reference genome by Bismark (ver. 0.14.5) [92]. After filtering the duplicate reads, the methylation information for each cytosine site was extracted. Methylation states were evaluated based on the binomial test followed by Benjamini–Hochberg false discovery rate (FDR < 0.01) correction, as described previously [99]. In the binomial test, the non-conversion rate was used as the expected probability. Only sites that covered more than four mapped reads were considered. The weighted methylation level was computed following the previously reported method [100].

### DMR detection

DMRs were identified using Metilene (ver. 0.2–6) [60]. For the domestication process, we compared methylome data between the wild soybean and landrace populations. For the improvement process, we compared methylome data between the landrace and cultivar populations. The accessions from each population were considered as repeats. A DMR was required to contain at least eight cytosine sites with < 300 bp in distance between adjacent cytosine sites. CG-DMR candidate regions, CHG-DMR candidate regions, and CHH-DMR candidate regions were required to have average methylation level differences of > 0.4, >0.4, and >0.2 between the corresponding populations. Finally, the regions with Bonferroni correction q-value < 0.01 were determined as DMRs.

### DSR resources

All the original DSRs were downloaded from the previous study [16]. Soybean reference genome v189 was used in that study and the new reference genome v275 was used in this study. To reconcile the genomic positions, we converted the DSRs from v189 to v275 reference using Blast+ [101] and Mummer (ver. 3.0) [102].

### siRNA cluster identification

The small RNA-seq reads were quality controlled by FastQC [103] and reads from different accessions were combined for siRNA cluster analysis using the ShortStack pipeline (ver. 3.8.4) [104]. The mincov parameter was set as 450. The expression levels of the 24 nt siRNAs (reads per million, RPM) for each accession were calculated as follows: number of reads mapped to the siRNA cluster divided by total read number for the accession.

### TE variant detection

Soybean TE annotations were downloaded from SoyTEdb [105] for the v108 reference genome; these TEs were converted to the v275 reference genome by Blast+ [101]. TE variants were detected using TEPID, as described previously [72]. The average insert size was set to 280; all the other parameters were set as default.

### Local association study

We performed a local association study for DMRs using the methylation variation of each accession with the corresponding siRNA expression, TE presence/absence, and SNPs in this region. To associate the overlapping DMR/siRNA pairs and the nearest DMR/TE-var pairs, Pearson correlation was applied. To test the significance of each pairwise correlation, bootstrap correlation coefficient estimates were collected based on 1000 permutations of the accession names. DMR/siRNA and DMR/TE-var associations were deemed significant if they had a correlation coefficient higher than those of all 1000 permutations (*p* < 1/1000). The local association between DMRs and their nearby SNPs were analyzed as Eichten et al. described previously [43].

### Gene expression and functional analysis

After removing the reads with low quality and clipping the adapter sequences by Trimmomatic (ver. 0.36) [98], the raw RNA sequence data for each accession were mapped to the corresponding pseudo-reference genome using HISAT2 (ver. 2.0.4) [106]. Gene expression was estimated using StringTie (ver. 1.3.1) [107] and normalized using the numbers of reads per kilobase of exon sequence in a gene per million mapped reads (FPKM). GO analysis was performed using agriGO (ver. 2.0) [108] and KEGG pathway analysis was performed using KOBAS 3.0 [109]. GO terms and pathways that contained > 5 analysis genes with enrichment q-values < 0.05 were considered significantly enriched.

## Additional files

Additional file 1: **Table S1.** Information of sequenced accessions. (XLSX 11 kb)

Additional file 2: **Table S2.** DNA-seq mapping and SNP calling for sequenced accessions. (XLSX 12 kb)

Additional file 3: **Figure S1.** Pipeline for WGBS analysis. **Figure S2.** Genetic diversity difference among different genomic regions. **Figure S3.** Genetic diversity comparison between DMR, DSR, and NSR in wild, landrace, and cultivar populations. **Figure S4.** The genetic diversity changes between corresponding populations for increased and decreased DMRs. **Figure S5.** The genetic diversity comparisons between corresponding populations for domestication DMRs (a) and improvement DMRs (b). **Figure S6.** Overlap between domestication and improvement DMRs for different cytosine contexts. **Figure S7.** Relationship of CG and CHG methylation levels for overlapped CG-DMRs and CHG-DMRs. **Figure S8.** Length comparisons among overlapped CG-DMRs and CHG-DMRs, unique CG-DMRs, and unique CHG-DMRs. **Figure S9.** Hierarchical clustering of methylation level and corresponding siRNA expression for associated DMR/siRNA pairs in domestication (a) and improvement (b). **Figure S10.** The genetic diversity difference between TE variant associated DMRs and TE variant not-associated DMRs for different genomic regions. **Figure S11.** Overlap between overlapped CG-DMRs and CHG-DMRs (O-CG/CHG DMRs) who associated with genetic variations for domestication (a) and improvement (b). **Figure S12.** Overlap and GO enrichment analysis for genes in “pure Dos_CG-DMRs” and “pure Dos_CHG-DMRs.” **Figure S13.** The correlation between CG methylation level and expression level for genes in “pure DMRs.” (PDF 3689 kb)

Additional file 4: **Table S3.** WGBS mapping for sequenced accessions. (XLSX 14 kb)

Additional file 5: **Table S4.** Methylated cytosine site statistics for each sequenced accession. (XLSX 14 kb)

Additional file 6: **Table S5.** List of detected DMRs. (XLSX 403 kb)

Additional file 7: **Table S6.** List of locally associated DMR/siRNA pairs. (XLSX 26 kb)

Additional file 8: **Table S7.** List of locally associated DMR/TE-variance pairs. (XLSX 20 kb)

Additional file 9: **Table S8.** List of locally associated DMRs and nearby SNPs. (XLSX 45 kb)

Additional file 10: **Table S9.** GO and KEGG pathway enrichments of different “pure DMR” types. (XLSX 9 kb)

Additional file 11: **Table S10.** List of “pure Dos_CG-DMR” overlapping genes encoding enzymes involved in carbohydrate metabolism pathways. (XLSX 11 kb)

## Abbreviations

ANOVA: Analysis of variance
DMR: Differentially methylated region
Dos: Domestication
DSR: DNA sequence regions under selection
GO: Gene Ontology
GWAS: Genome-wide association study
Imp: Improvement
KEGG: Kyoto Encyclopedia of Genes and Genomes
MVW: Methylation variation window
NSR: Non-selected region
o-CG/CHG-DMR: Overlapping CG-DMRs and CHG-DMR
QTL: Quantitative trait loci
siRNA: Small interfering RNA
SNP: Single-nucleotide polymorphisms
TE: Transposable element
u-CG-DMR: Unique CG-DMR
u-CHG-DMR: Unique CHG-DMR
WGBS: Whole-genome bisulfite sequencing
