# Supplementary material for: DNA methylation footprints during soybean domestication and improvement
Source: Genome Biol. 2018 Sep 10;19:128. doi: 10.1186/s13059-018-1516-z (PMC6130073; doi:10.1186/s13059-018-1516-z)
Supplement: Supplementary file 3 — Figure S1. Pipeline for WGBS analysis. Figure S2. Genetic diversity difference among different genomic regions. Figure S3. Genetic diversity comparison between DMR, DSR, and NSR in wild, landrace, and cultivar populations. Figure S4. The genetic diversity changes between corresponding populations for increased and decreased DMRs. Figure S5. The genetic diversity comparisons between corresponding populations for domestication DMRs (a) and improvement DMRs (b). Figure S6. Overlap between domestication and improvement DMRs for different cytosine contexts. Figure S7. Relationship of CG and CHG methylation levels for overlapped CG-DMRs and CHG-DMRs. Figure S8. Length comparisons among overlapped CG-DMRs and CHG-DMRs, unique CG-DMRs, and unique CHG-DMRs. Figure S9. Hierarchical clustering of methylation level and corresponding siRNA expression for associated DMR/siRNA pairs in domestication (a) and improvement (b). Figure S10. The genetic diversity difference between TE variant associated DMRs and TE variant not-associated DMRs for different genomic regions. Figure S11. Overlap between overlapped CG-DMRs and CHG-DMRs (O-CG/CHG DMRs) who associated with genetic variations for domestication (a) and improvement (b). Figure S12. Overlap and GO enrichment analysis for genes in “pure Dos_CG-DMRs” and “pure Dos_CHG-DMRs.” Figure S13. The correlation between CG methylation level and expression level for genes in “pure DMRs.” (PDF 3689 kb) [file 13059_2018_1516_MOESM3_ESM.pdf]

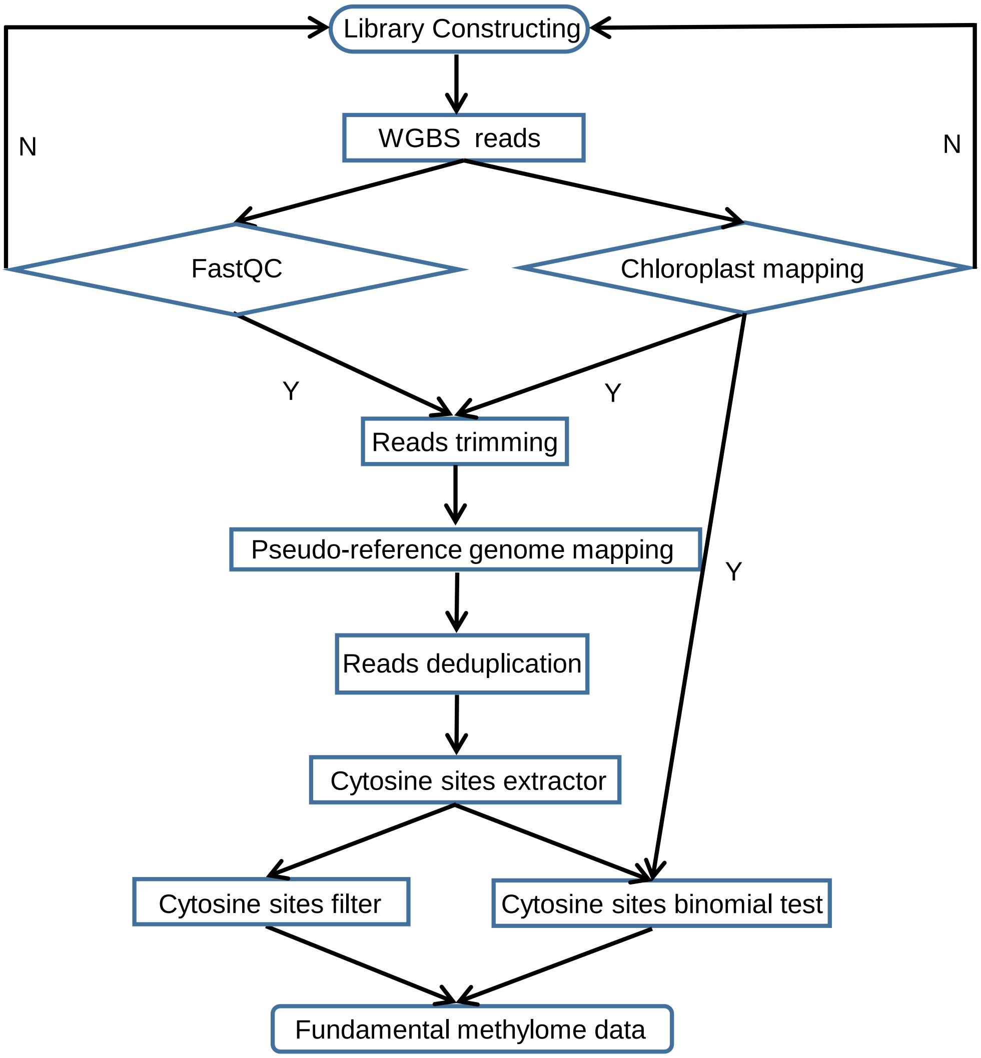

Figure S1. Pipeline for WGBS analysis

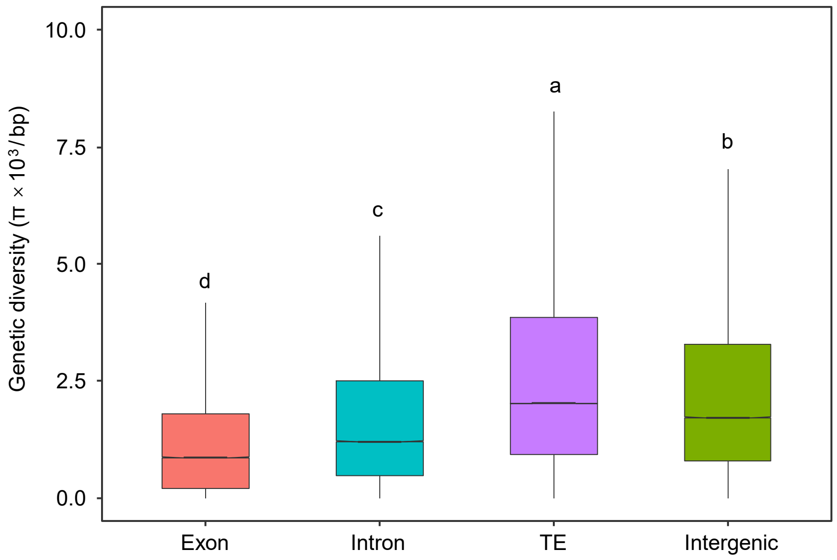

Figure S2. Genetic diversity difference among different genomic regions. To have a statistical significance, we divided soybean genome into continuous 500bp windows and only windows overlapped more than 80% by specific genomic region type were retained for this analysis. The different letters on top of each column indicate significant differences in ANOVA test ($p<0.01$).

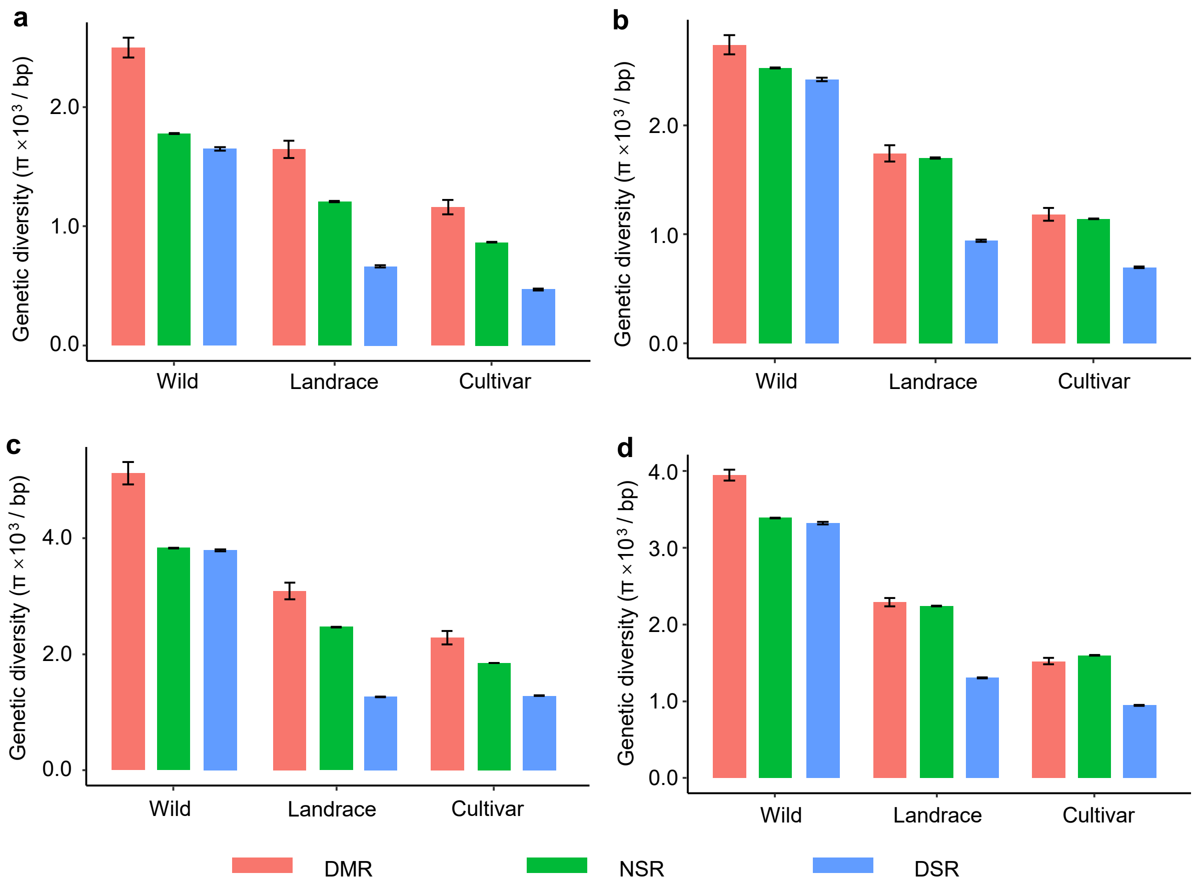

Figure S3. Genetic diversity comparison between DMR, DSR and NSR in wild, landrace and cultivar populations. a Exon region. **b** Intron region. **c** TE region. **d** Intergenic region.

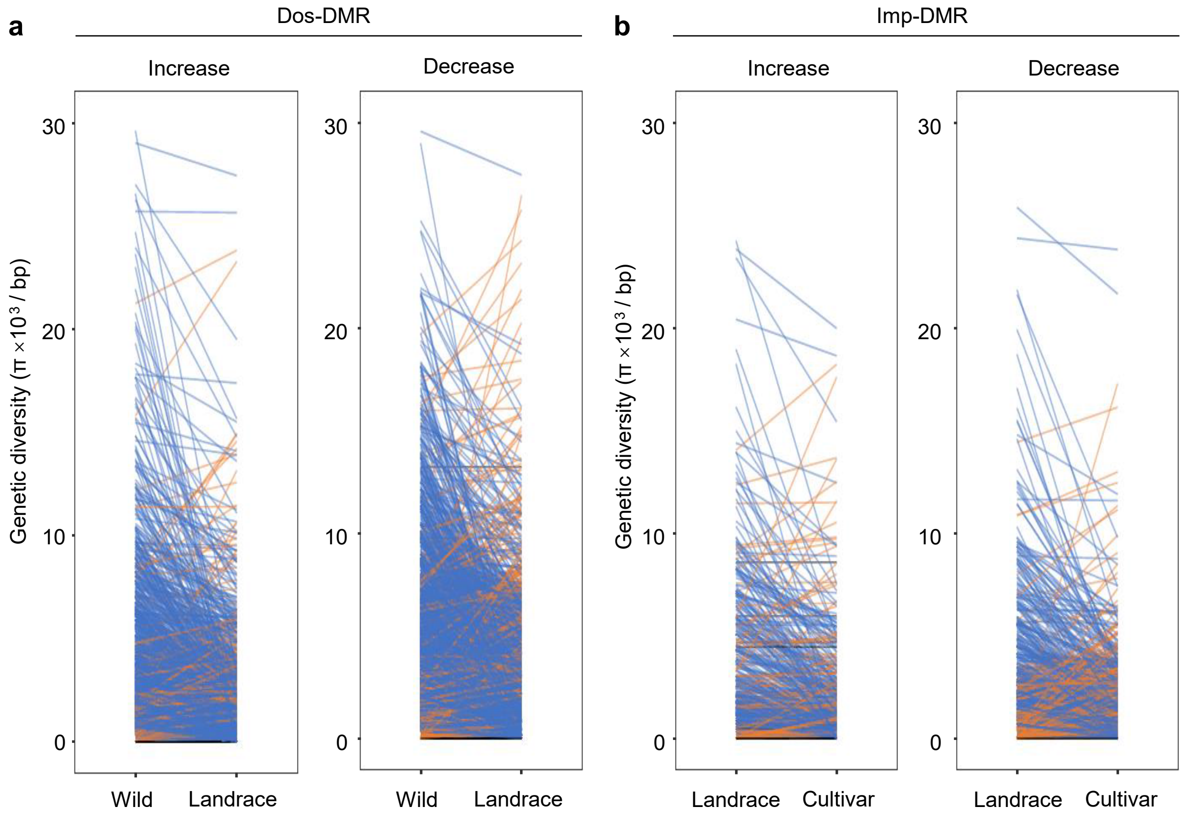

Figure S4. The genetic diversity changes between corresponding populations for increased and decreased DMRs. a Genetic diversity changes in domestication process. **b** Genetic diversity changes in improvement process. Each line in the panels indicates one DMR.

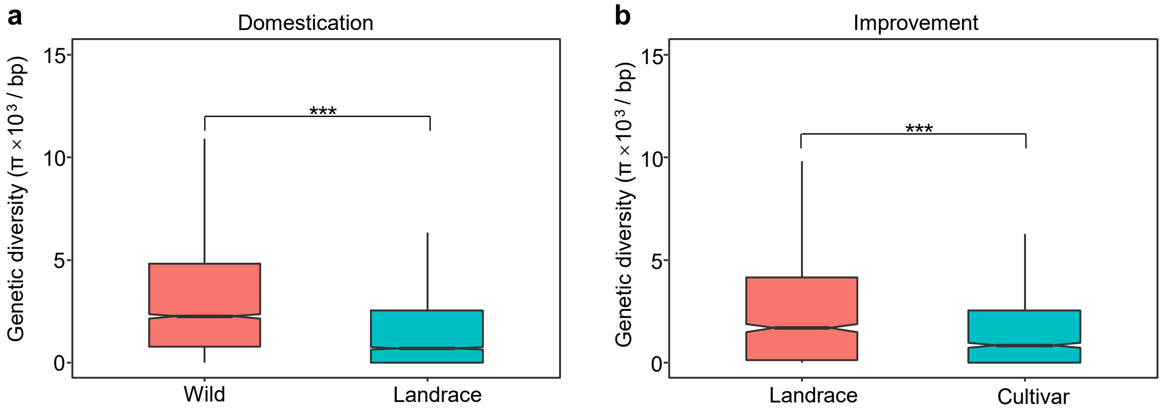

Figure S5. The genetic diversity comparisons between corresponding populations for domestication DMRs (a) and improvement DMRs (b). *** denoted t-test $p<0.001$.

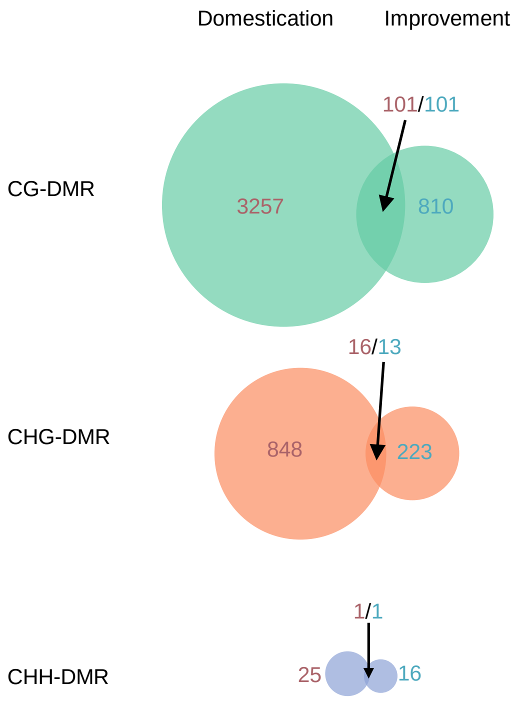

Figure S6. Overlap between domestication and improvement DMRs for different cytosine contexts.

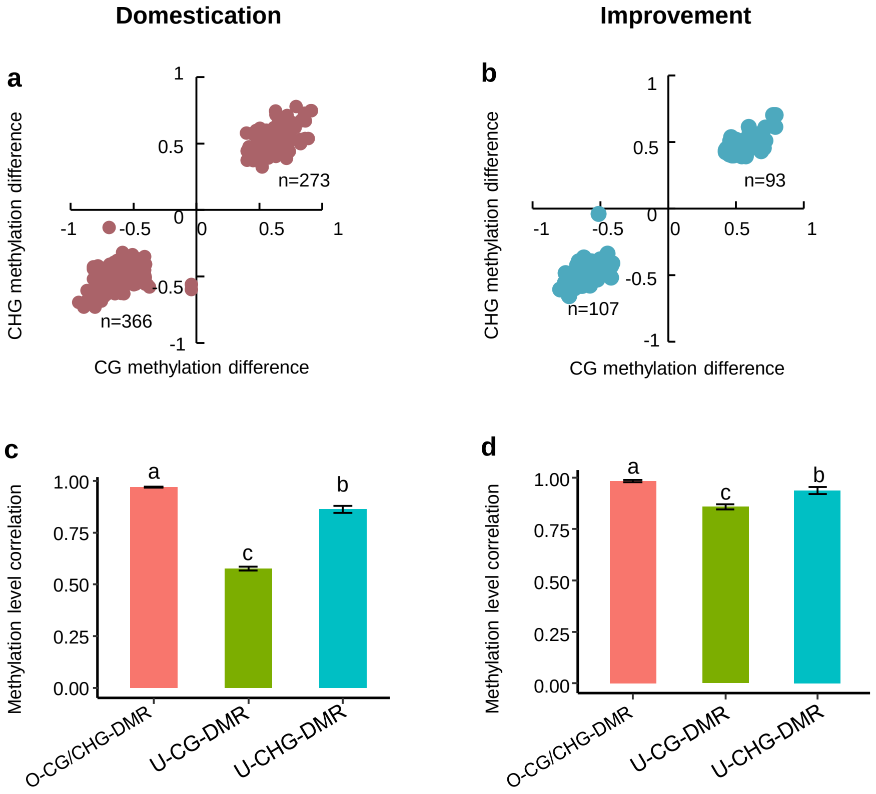

Figure S7. Relationship of CG and CHG methylation levels for overlapped CG-DMRs and CHG-DMRs. a CG methylation difference (x axis) and CHG methylation difference (y axis) between wild and landrace (wild-landrace) from domestication process. **b** CG methylation difference (x axis) and CHG methylation difference (y axis) between landrace and cultivar (landrace-cultivar) from improvement process. **c** Pearson correlation for CG and CHG methylation level in overlapped CG-DMRs and CHG-DMRs, unique CG-DMRs and unique CHG-DMRs from domestication process. **d** Pearson correlation for CG and CHG methylation level in overlapped CG-DMRs and CHG-DMRs, unique CG-DMRs and unique CHG-DMRs from improvement process. The different letters on top of each column indicate significant differences in ANOVA test (p<0.01). The bars indicate standard error.

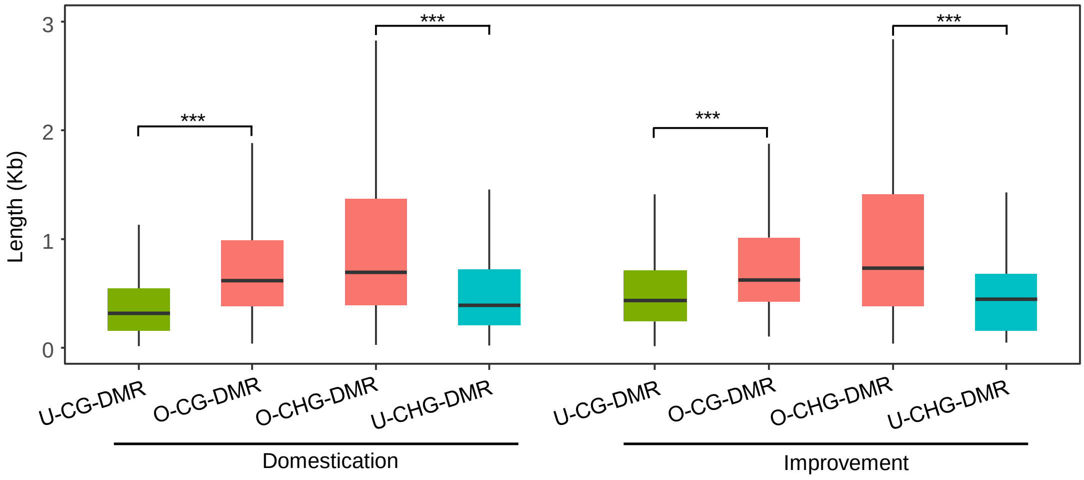

Figure S8. Length comparisons among overlapped CG-DMRs and CHG-DMRs, unique CG-DMRs and unique CHG-DMRs. *** denoted t-test $p<0.001$.

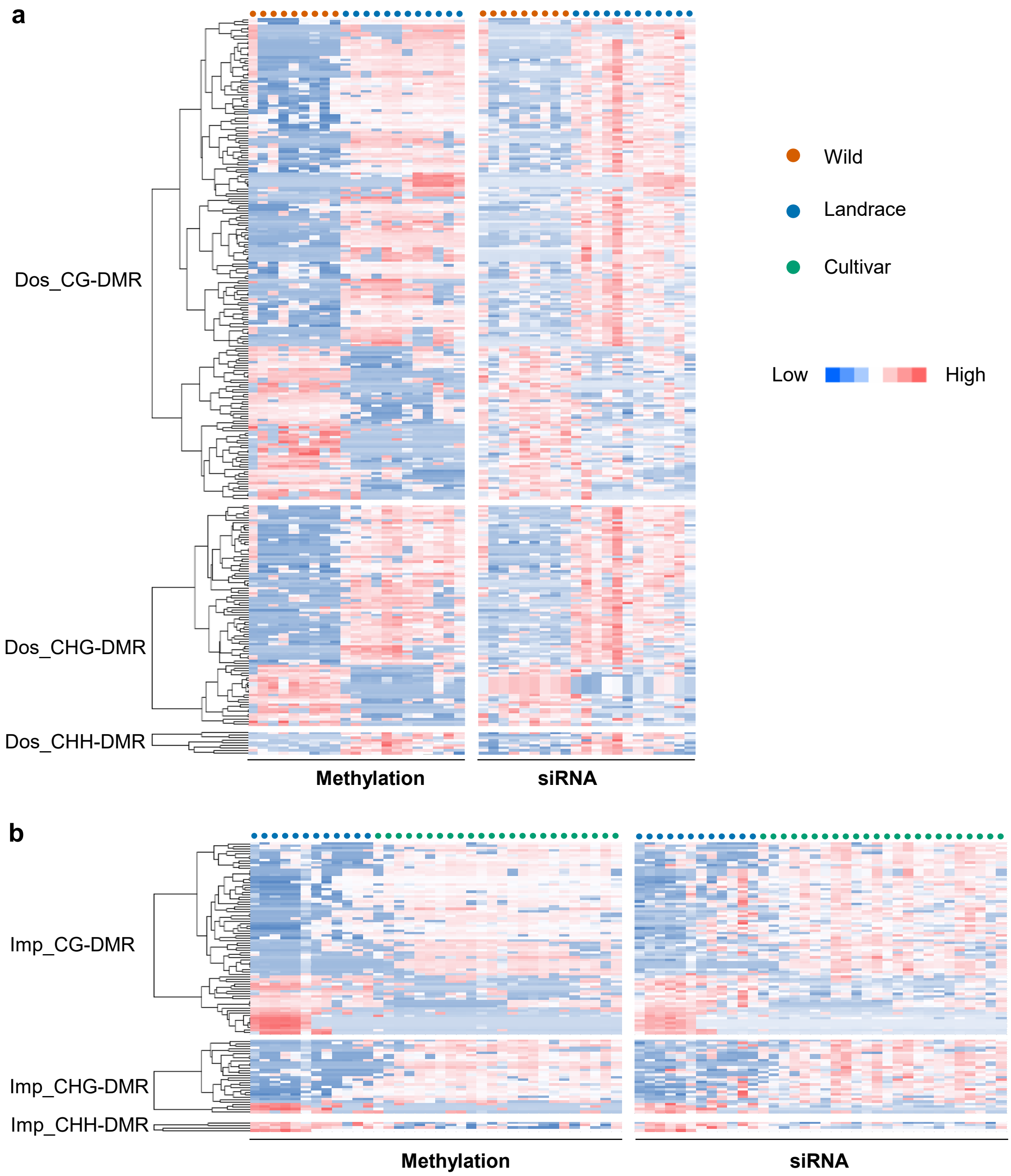

Figure S9. Hierarchical clustering of methylation level and corresponding siRNA expression for associated DMR/siRNA pairs in domestication (a) and improvement (b).

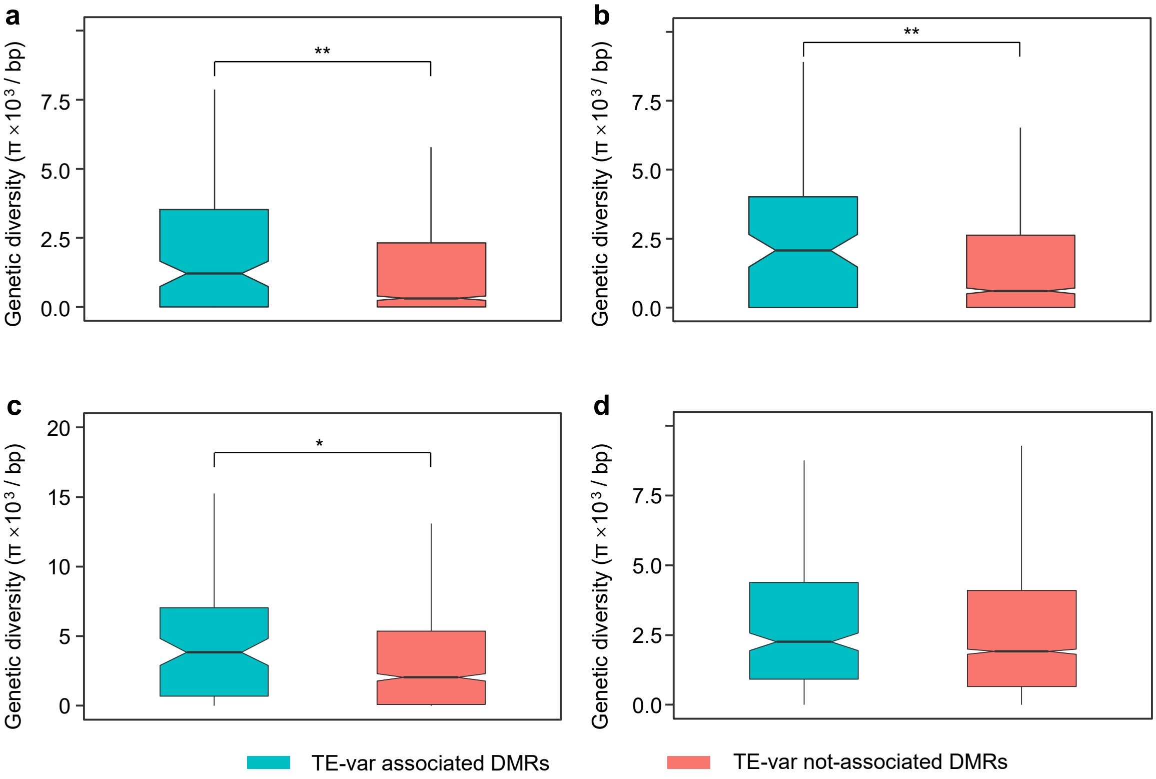

Figure S10. The genetic diversity difference between TE variant associated DMRs and TE variant not-associated DMRs for different genomic regions. a Exon region. **b** Intron region. **c** TE region. **d** Intergenic region. * denoted t-test $p<0.05$, ** denoted t-test $p<0.01$.

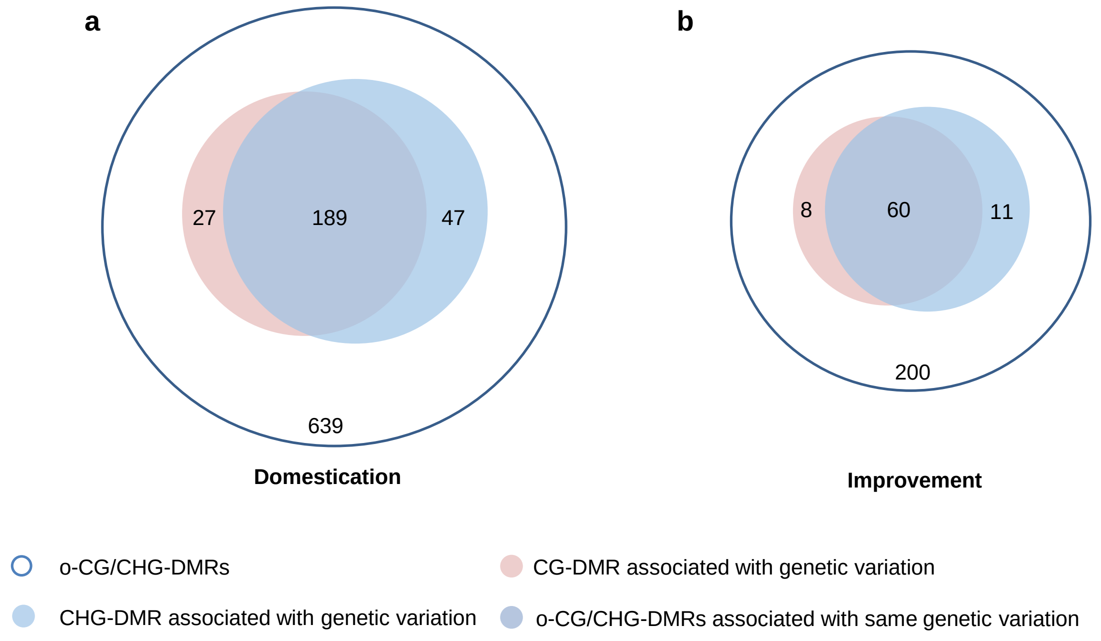

Figure S11. Overlap between overlapped CG-DMRs and CHG-DMRs (O-CG/CHG DMRs) who associated with genetic variations for domestication (a) and improvement (b).

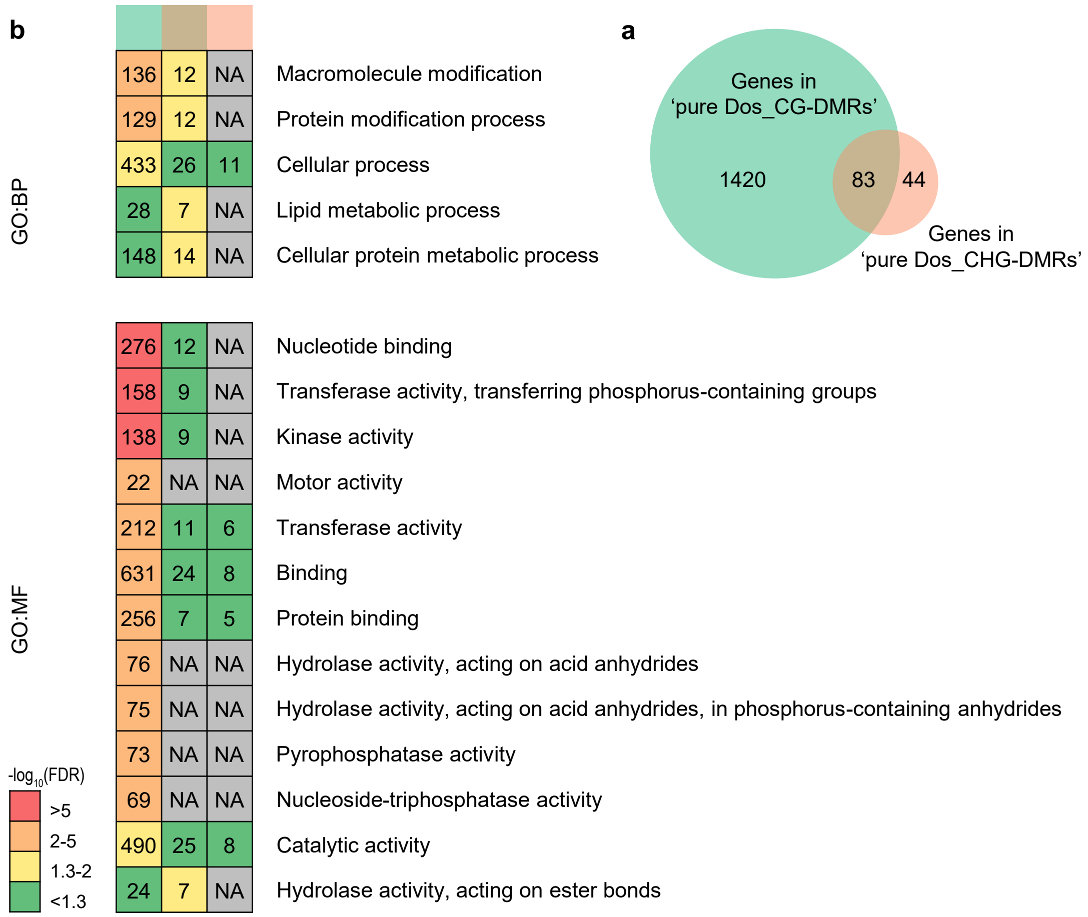

Figure S12. Overlap and GO enrichment analysis for genes in 'pure Dos_CG-DMRs' and 'pure Dos_CHG-DMRs'. a The overlap genes in 'pure Dos_CG-DMRs' and in 'pure Dos_CHG-DMRs'. **b** GO enrichment analysis of the overlapped and non-overlapped genes. The number in each cell is the gene number belonging to corresponding GO term. GO terms contained more than 5 analyzed genes and enrichment q value less than 0.05 are considered as significant enrichment; GO:BP denotes GO terms for biological process and GO:MF denotes GO terms for molecular function.

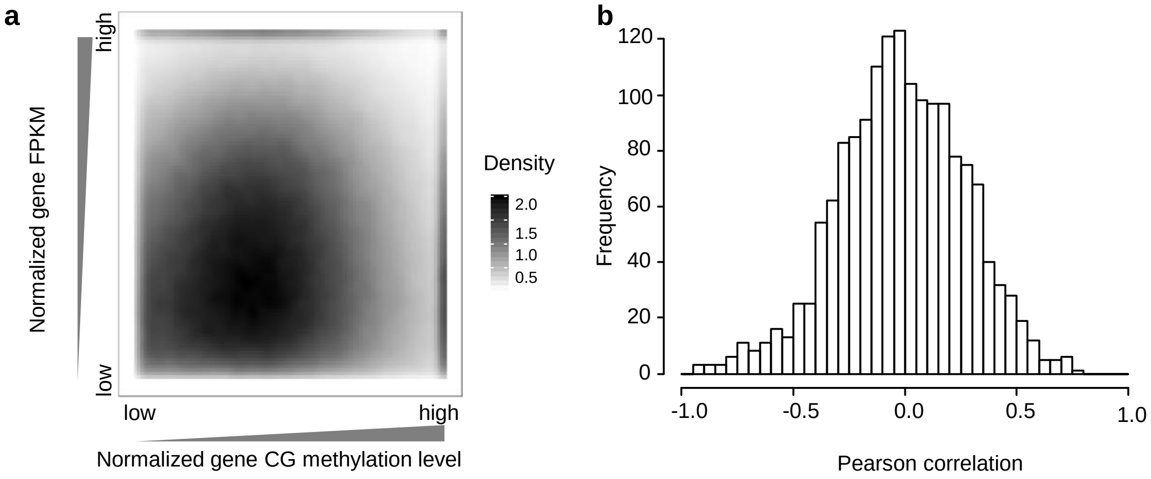

Figure S13. The correlation between CG methylation level and expression level for genes in 'pure DMRs'. a Density plot for scale normalized gene CG methylation level (x axis) and scale normalized expression value (y axis). **b** Histogram of Pearson correlation coefficient between gene CG methylation level and expression level.
